# Automated segmentation of neurons and spinal cord structures in immunofluorescence images using SpineDL

**DOI:** 10.1038/s41598-026-57519-w

**Published:** 2026-06-20

**Authors:** Pablo Ruiz-Amezcua, Daniel Franco-Barranco, David Reigada, Teresa Muñoz-Galdeano, Rodrigo M. Maza, Manuel Nieto-Diaz

**Affiliations:** 1 Hospital Nacional de Parapléjicos (SESCAM), Toledo, Spain; 2 Department of Experimental Biology, University of Jaén, Jaén, Spain; 3 Instituto de Investigación Sanitaria de Castilla-La Mancha (IDISCAM), Toledo, Spain; 4 MRC Laboratory of Molecular Biology, Cambridge, UK; 5 Department of Physiology, Development and Neuroscience, University of Cambridge, Cambridge, UK; 6 Donostia International Physics Center (DIPC), San Sebastián, Spain

**Keywords:** Spinal cord injury, Mouse models, Instance segmentation, Semantic representations, Histology, Biological techniques, Computational biology and bioinformatics, Neuroscience

## Abstract

In this study, we present SpineDL, an open-source deep learning (DL) approach for neuron and anatomical structure segmentation of the spinal cord in fluorescence images immunostained with NeuN and DAPI, within the context of murine models of spinal cord injury (SCI). SpineDL comprises two main modules: SpineDL-Neuron, for instance-level identification of neuronal somas; and SpineDL-Structure, for semantic segmentation of key spinal cord structures including gray matter, white matter, ependyma, and damaged tissue. To train the models, we developed the SpineDL dataset, a curated collection of 161 confocal images of mouse spinal cord, manually annotated by SCI researchers and organized into specific subsets. Both models are based on the HRNetV2-W64 architecture and were trained using state-of-the-art data augmentation and optimization techniques, implemented within the BiaPy framework, following an iterative refinement process driven by quantitative evaluation, SCI researcher feedback, and systematic error analysis. Our results demonstrate that SpineDL achieves researcher-level performance in both structural segmentation and neuron identification tasks, showing high robustness across anatomical regions and injury conditions. Overall, this work provides a reproducible and extensible platform for quantitative analysis of neuron distribution in the naïve and injured spinal cord, supporting automation, standardization, and scalability of histopathological workflows in neuroscience research and preclinical studies and translational applications.

## Introduction

Spinal cord injury (SCI) affects an estimated 15 million people worldwide, leading to permanent motor, sensory, and autonomic deficits with substantial personal, medical, and economic consequences, estimated at $1.5–6.2 million per patient in the USA alone^1^. Despite decades of research, no treatment has yet demonstrated sufficient efficacy and security to enter routine clinical use^2^.

This limited therapeutic progress reflects the extreme biological complexity of SCI pathophysiology, in which different primary insults trigger a highly dynamic cascade of secondary events including vascular disruption, inflammatory responses with infiltration of immune cell populations, multiple forms of regulated cell death, and progressive changes in the extracellular and cellular microenvironment. SCI processes and events affect a highly diverse pool of specialized neuronal and non-neuronal populations and evolve differently across injuries, space, time, and individuals ^3^. Technological advances such as single-nucleus RNA sequencing and spatial transcriptomics have greatly improved our ability to analyse this complexity and, in doing so, have demonstrated the importance of spatially resolved cellular analysis for understanding SCI pathophysiology and developing effective therapies. For example, the combination of single-nucleus RNA sequencing and spatial transcriptomics have identified a population of excitatory interneurons in the intermediate laminae of the lumbar spinal cord that is required for locomotor recovery during epidural electrical stimulation after SCI ^4^, while related spatial transcriptomic strategies have identified supraspinal glutamatergic populations in the lateral hypothalamus, whose activation enhances walking recovery after SCI ^5^. These analyses have focused particularly on neurons because of their central role in spinal cord function and their remarkable diversity in connectivity, neurotransmitter identity, molecular profile, and functional specialization.

Histology remains the main source of cellular and spatial information in SCI research and routinely generates stained sections from thousands of animals across laboratories. These materials capture spinal neuron distribution across diverse conditions and can provide key insights into neuroanatomical–functional and pathophysiological relationships, as well as help identify therapeutic targets. However, the extraction of such information has classically relied on manual segmentation, which is both time-consuming, error-prone, and difficult to scale for the large datasets required to capture the spatial complexity of injured spinal cord ^6^.

Over the past decade, deep learning (DL) has driven significant advancements in computer vision with applications for the analysis of SCI. DL-based approaches have enabled automated analysis of MRI images in humans^7–10^, as well as SCI classification based on electromyographic data in nonhuman primates^11^. In this context, nnU-Net has emerged as a widely adopted baseline due to its self-configuring design, which automatically adapts preprocessing, architecture, and training strategies to each dataset^12^. Accordingly, many spinal cord segmentation methods—such as TotalSpineSeg, SCIseg, and gmseg^13^—are built upon similar frameworks. However, these approaches are primarily designed for MRI data and are therefore not directly transferable to fluorescence histology. In preclinical and basic settings, particularly in murine models, DL techniques have been increasingly employed to microscopy data. These include segmentation of axons and myelin in electronic and light microscopy images^10,11,14^ as well as the identification of neuronal somas in multiphoton, 3D fluorescence, and other optical imaging modalities^15–17^. Such approaches have significantly enhanced throughput and reproducibility compared to manual or threshold-based analyses. In parallel, general-purpose bioimage analysis tools such as Cellpose ^18^, StarDist ^19^, Ilastik ^20^ and BiaPy ^21^, together with their commercial equivalents, have substantially expanded access to automated segmentation across diverse microscopy applications^6^. However, tools specifically designed for automatic neuron segmentation in injured spinal cord tissue remain lacking.

In this study, we present SpineDL, an open-source deep learning-based tool designed for the segmentation of spinal cord neurons using fluorescence images immunostained against Rbfox3 (usually known as NeuN, a widely used marker of postmitotic CNS neurons), and DAPI (4’,6- diamidino- 2-phenylindole, a common fluorescent stain for nuclei) in the context of SCI. The tool has been developed using BiaPy^21^, an open-source library tailored for constructing deep learning workflows in bioimage analysis. SpineDL offers two main modules: (1) SpineDL Neuron, for automatic identification of neuron somas; and (2) SpineDL-Structure, for automatic segmentation of key anatomical structures of the spinal cord, including the gray matter, white matter, ependyma, and damaged regions. This second module was incorporated to simplify anatomical representation and facilitate downstream image registration and spatial reconstruction ^22,23^.

In parallel with the development of the tool, we introduce the SpineDL dataset, a curated collection of confocal fluorescence images of mouse spinal cord sections labeled with NeuN and DAPI. The dataset compiles images from two previously published studies^24,25^ employing murine models of traumatic SCI and includes comprehensive manual annotations for both spinal cord structures and neuronal somas. These annotations were generated through iterative refinement involving deep neural network feedback and researcher review, resulting in two high-quality subsets: one for neuron identification and another for structure segmentation. The final dataset, comprising 161 annotated images across training, validation, and test sets, is publicly available through Zenodo, providing a valuable resource for benchmarking and advancing deep learning approaches in spinal cord histopathology.

The aim of SpineDL is to provide an accessible, flexible, and reproducible platform for spatial analysis of neuronal death in SCI models. The tool enables automated segmentation of spinal cord neurons and structures before and after injury. This workflow facilitates the study of injury associated anatomical changes and offers a robust framework for investigating cellular mechanisms underlying SCI pathophysiology.

We summarize our main contributions as follows:

- Introduces SpineDL, a novel open-source deep learning-based tool designed specifically for automated segmentation and analysis of spinal cord fluorescence images in the context of spinal cord injury.
- Releases the SpineDL dataset, a publicly available, expertly annotated collection of fluorescence images with neuron soma annotations and structure labels, providing a standardized benchmark for developing and validating deep learning methods in spinal cord histology.

## Methods

### SpineDL dataset

#### Dataset acquisition

The images analyzed in this study were sourced from two independently published investigations employing mouse models of traumatic spinal cord injury. The first,^25^ examined cell-specific alterations in autophagy following contusive SCI. This heterogeneous dataset includes spinal cord sections from female mice with and without spinal cord injury, collected across independent studies. Samples were obtained at different post-injury time points and rostrocaudal positions relative to the lesion, and include multiple immunofluorescent co-labeling combinations in addition to NeuN and DAPI. The second,^24^ evaluated the neuroprotective effects of the anti-apoptotic drug ucf-101. In contrast to the first study, this dataset corresponds to a controlled preclinical trial with three experimental conditions, in which animals, injury procedures, collection time, staining protocols, and imaging conditions were highly homogeneous. In both studies, confocal microscopy images were acquired from 20 ${\upmu }$m transverse sections of the thoracic spinal cord of adult mice (*Mus musculus*, strain C57BL/6). Sections were fluorescently stained with two routine markers: an antibody against NeuN to label neuronal somas and DAPI as a general nuclear counterstain.

Full spinal cord sections were originally imaged as mosaics of tiled images using either a High-Speed Resonant Confocal Scanner Microscope or a TCS SP5 fast scan confocal microscope (Leica Microsystems CMS GmbH,Wetzlar, Germany), both equipped with PL APO 20*×*/0.70 CS dry UV objectives. The original raw images were acquired as Z-stacks at varying resolutions from 0.445 to 0.891 *μ*m/pixel with 8 to 32-bit depth and stored in .lif files. Preprocessing included maximum intensity projection of the stacks, the arrangement of the images into two grayscale channels (Channel 1: DAPI; channel 2: NeuN) and their conversion to TIFF format. Additionally, some images were cropped for training on specific features. All preprocessing was carried out using Fiji ^26^.

Images were split into training, validation, and testing subsets, ensuring a balanced representation of images from each of the original studies. In total, the dataset comprises 161 images. Of these, 49 images correspond to full spinal cord sections from 19 individuals of the autophagy study^25^ . The remaining 112 images derive from the preclinical analysis of ucf-101^24^ and correspond to 77 spinal cord sections from 13 individuals, including 60 full-section images and 47 cropped images from 17 additional sections used to focus on specific local structures of interest, particularly artifacts such as the meninges, allowing efficient annotation of fine-grained features of the Neuron identification model.

The test sets were defined a priori to ensure strict independence from the training process while capturing a broad range of biological and experimental variability. To this end, five images were selected from each of the two source studies, encompassing diverse injury conditions, staining protocols, and imaging characteristics.

Images and metadata—including source study, individual, condition, section, and staining—are accessible in our Zenodo repository.

#### Dataset annotation

Given the different nature of the tasks–instance-level neuron identification versus semantic segmentation of anatomical structures–the two datasets were constructed through related but task-specific iterative strategies.

**Fig. 1:**
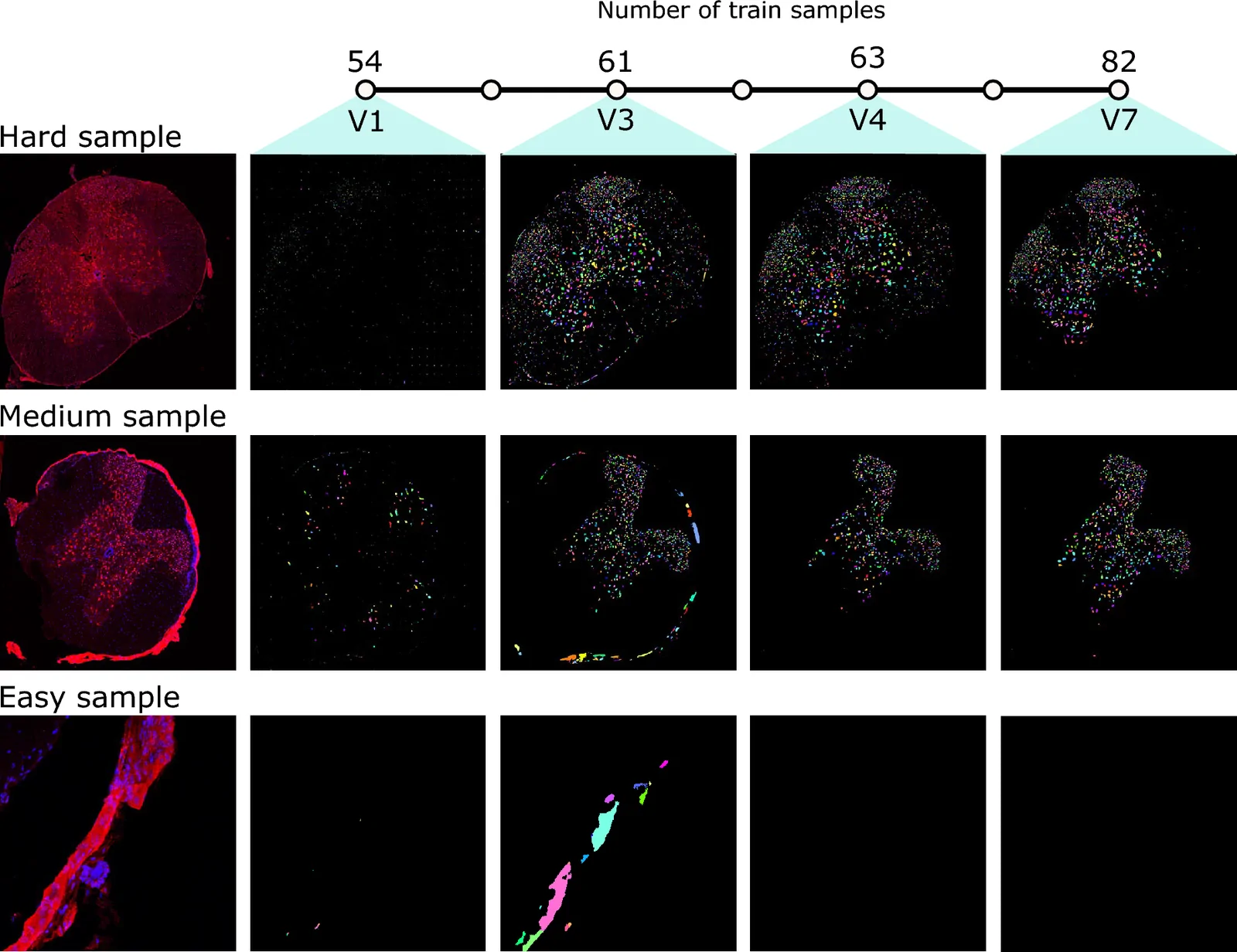
Overview of the process used to construct the neuron identification dataset. This process underwent seven iterations. The first column displays selected raw images and the remaining columns show the segmentations produced by the DNN across the different iterations. The process involved refining the labels generated by the DNN in previous iterations and gradually increasing the number of training samples. The figure shows three representative examples categorized as easy, medium, and hard, based on the level of difficulty the DNN encountered in generating accurate segmentations. As illustrated, the DNN’s performance improves steadily across iterations. V1, V3, V4, and V7 indicate the DNN versions obtained after 1, 3, 4, and 7 iterations, respectively.

**Neuron identification dataset**. This dataset was constructed through seven controlled iterations (Figure 1), progressively expanding the training set and refining segmentation masks from previous rounds. At each iteration, new training samples were selected based on low-performing regions identified through visual inspection by spinal cord injury (SCI) researchers, specifically targeting areas with artifacts or systematic segmentation errors.

A key challenge was the DNN’s tendency to incorrectly identify neurons in regions affected by staining artifacts (e.g., meninges depicted in the “medium sample” of Fig. 1), which do not contain neurons. To address this issue, representative image patches containing such artifacts were explicitly incorporated into the training set, enabling the model to distinguish non-neuronal structures. The final dataset comprises 82 images (75 training, 7 validation).

For evaluation, the test set (n = 10) combines two complementary sources. The first subset (n = 5), from the ucf-101 preclinical analysis^24^, corresponds to a previously established benchmark for assessing precision, repeatability, and reproducibility of neuron identification methods^6^. To ensure representation of both data sources used during training, a second subset (n = 5) from the study of autophagy in SCI^25^ was included. Images were selected to cover a broad range of conditions, from uninjured tissue with clear neuronal staining to injured sections with strong background signal. Each test image was independently annotated in five sessions by three SCI researchers (two performing replicate analyses), marking all detected neuronal candidates. Annotations were merged, and each object was assigned a likelihood score defined as the fraction of annotations in which it was identified (out of five), providing a consensus-based ground truth.

In parallel, the DNN architecture and training configuration were iteratively adapted with the objective of achieving performance comparable to, or exceeding, expert-level agreement. The explored configurations and hyperparameters are detailed in the supplementary material (Table S2). The final model, trained on the fully refined dataset (iteration 7) and optimized to handle staining artifacts and heterogeneous imaging conditions, is referred to as SpineDL-Neuron.

**Fig. 2:**
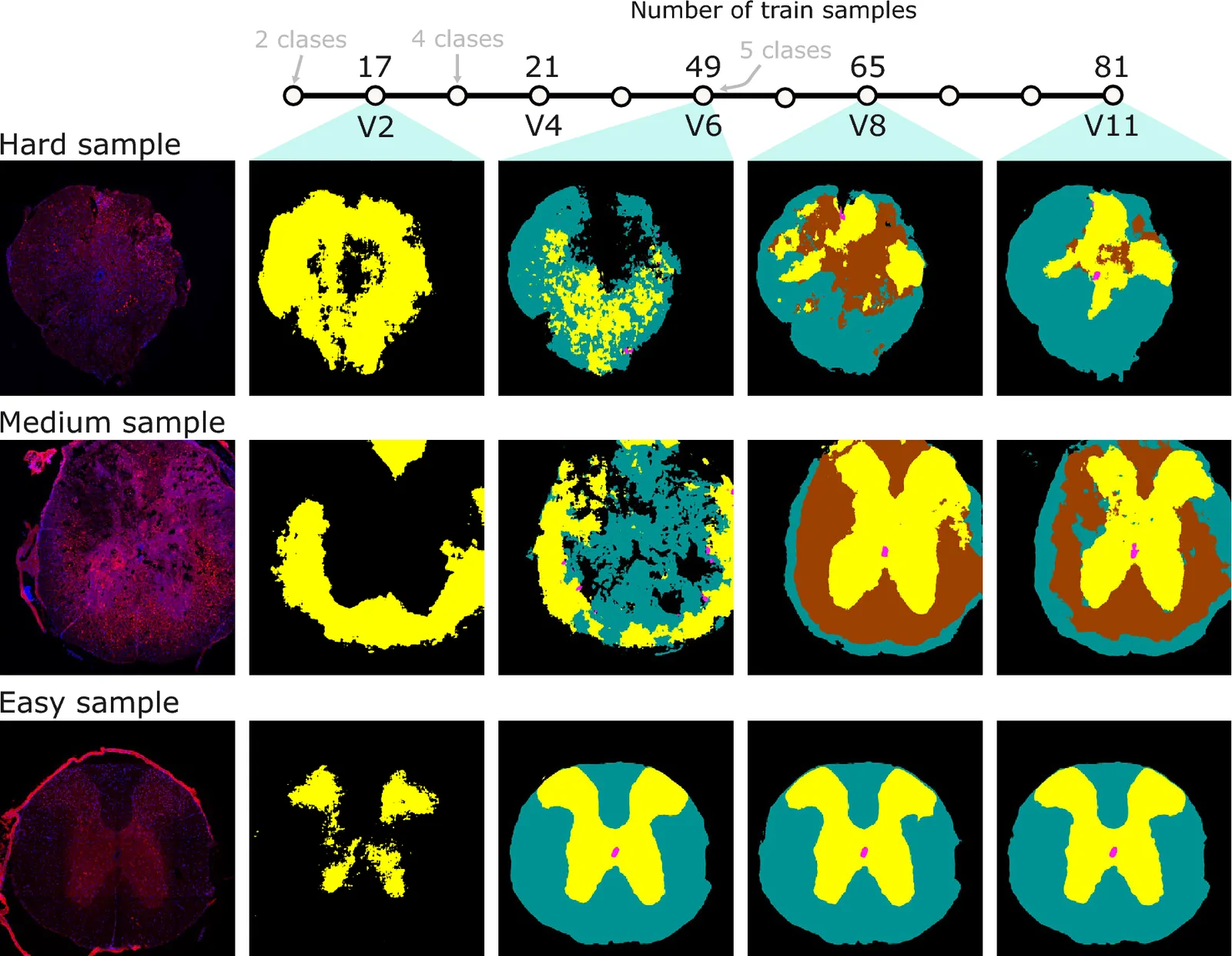
Overview of the process used to construct the structure identification dataset. This process underwent eleven iterations. The first column displays selected raw images and the remaining columns show the semantic segmentations produced by the DNN across the different iterations. The process involved refining the labels generated by the DNN in previous iterations and gradually increasing the number of training samples. Notice that for the creation of this data new classes were also added during the iterative process. The figure shows three representative examples categorized as easy, medium, and hard, based on the level of difficulty the DNN encountered in generating accurate segmentations. As illustrated, the DNN’s performance improves steadily across iterations. V2, V4, V6, V8, and V11 indicate the DNN versions obtained after 2, 4, 6, 8, and 11 iterations, respectively.

**Structure identification dataset**. The dataset was developed through eleven controlled iterations *(*Figure 2). The initial iteration (n = 10 images) included only gray matter and background, deliberately selected a priori as the minimal anatomical components required for downstream image registration and 3D reconstruction tasks. This simplified setup enabled an initial feasibility assessment despite the use of NeuN and DAPI stainings, which are primarily designed to label neuronal somas and cell nuclei rather than anatomical structures.

Subsequent iterations expanded the dataset by combining manual annotations with DNN-assisted labeling, in which model predictions were curated to improve efficiency. At each iteration, segmentation outputs were visually inspected by SCI researchers to assess prediction quality and guide dataset refinement. Once the network achieved reliable discrimination of spinal cord section and gray matter tissue, additional classes (white matter and ependyma) were incorporated (iteration 3).

To ensure robustness to biological and experimental variability, we followed a targeted data-engine strategy inspired by prior work (e.g., SAM ^27^ and CartoCell ^28^). The model was iteratively applied to new data, and samples exhibiting out-of-distribution characteristics (e.g., variations in contrast or morphology) were selectively added and manually corrected. This process was repeated across iterations to progressively improve generalization. Importantly, the test set was defined and strictly isolated prior to this iterative refinement, and was never used to guide model development, preventing data leakage.

By iteration 6 (n = 49), the model showed stable performance on non-injured tissue, enabling the inclusion of the most challenging class, injured tissue. Its annotation was inherently difficult due to the absence of damage-specific staining, requiring careful expert delineation. The final dataset (iteration 11) comprises 82 images (75 training, 7 validation) annotated across five classes: white matter, gray matter, ependyma, injured tissue, and background.

For the test set, a group of 3 independent SCI researchers annotated a set of 10 images from the two original studies, including intact and injured spinal cords, manually delineating the boundaries of the spinal cord section, excluding the dorsal root ganglia, the meninges and any obvious artifacts, as well as the gray matter, the ependyma, and the damaged tissue.

Model architectures and training configurations were co-optimized throughout the process with the explicit objective of maximizing performance while minimizing expert correction effort. The full set of explored hyperparameters and architectural variants is detailed in the supplementary material (Table S1). The final configuration, trained on the complete dataset and achieving performance within inter-expert variability, is referred to as SpineDL-Structure.

### Custom DNN architectures: 2D HRNetV2

Both DNNs, the one for neuron identification (SpineDL-Neuron) and the other for segmenting anatomical structures (SpineDL-Structure) follow the High-Resolution Network: HRNetV2-W64^29^. The DNN is a 2D convolutional architecture designed to preserve high-resolution representations throughout the entire feature extraction process. Unlike traditional encoder-decoder architectures that recover spatial details after successive downsampling, HRNet maintains multiple parallel branches operating at different resolutions and continuously exchanges information across them. This design enables the network to capture both fine-grained spatial information and robust contextual features. The W64 variant uses 64 channels in the high-resolution branch and progressively adds lower-resolution branches with increased channel capacities. Instance normalization and Leaky ReLU activations are applied after each convolutional layer to improve training stability and regularization.

Both models are fully integrated within the BiaPy library^21^, using Pytorch version 2.9.1. We used a patch size of *512× 512* for SpineDL-Neuron and *1280× 1280* in SpineDL-Structure. We employed data augmentation techniques such as, horizontal and vertical flips, brightness and contrast changes, elastic transformations and zoom variations. Cross entropy was used as a loss function in SpineDL-Neuron while cross entropy and Dice loss in SpineDL-Structure, with a learning rate of 1*e*–4, employing a cosine-decay scheduler with warm-up^30^, and ADAMW optimizer^31^. Both DNNs were trained until convergence using four NVIDIA GeForce RTX 3090.

Following the code of good practices to show deep learning-based results proposed by^32^, a thorough hyperparameter exploration throughout the SpineDL dataset creation of both models presented here, i.e. model SpineDL-Neuron and SpineDL-Structure, can be found in Table S1 and Table S2 respectively.

**Fig. 3:**
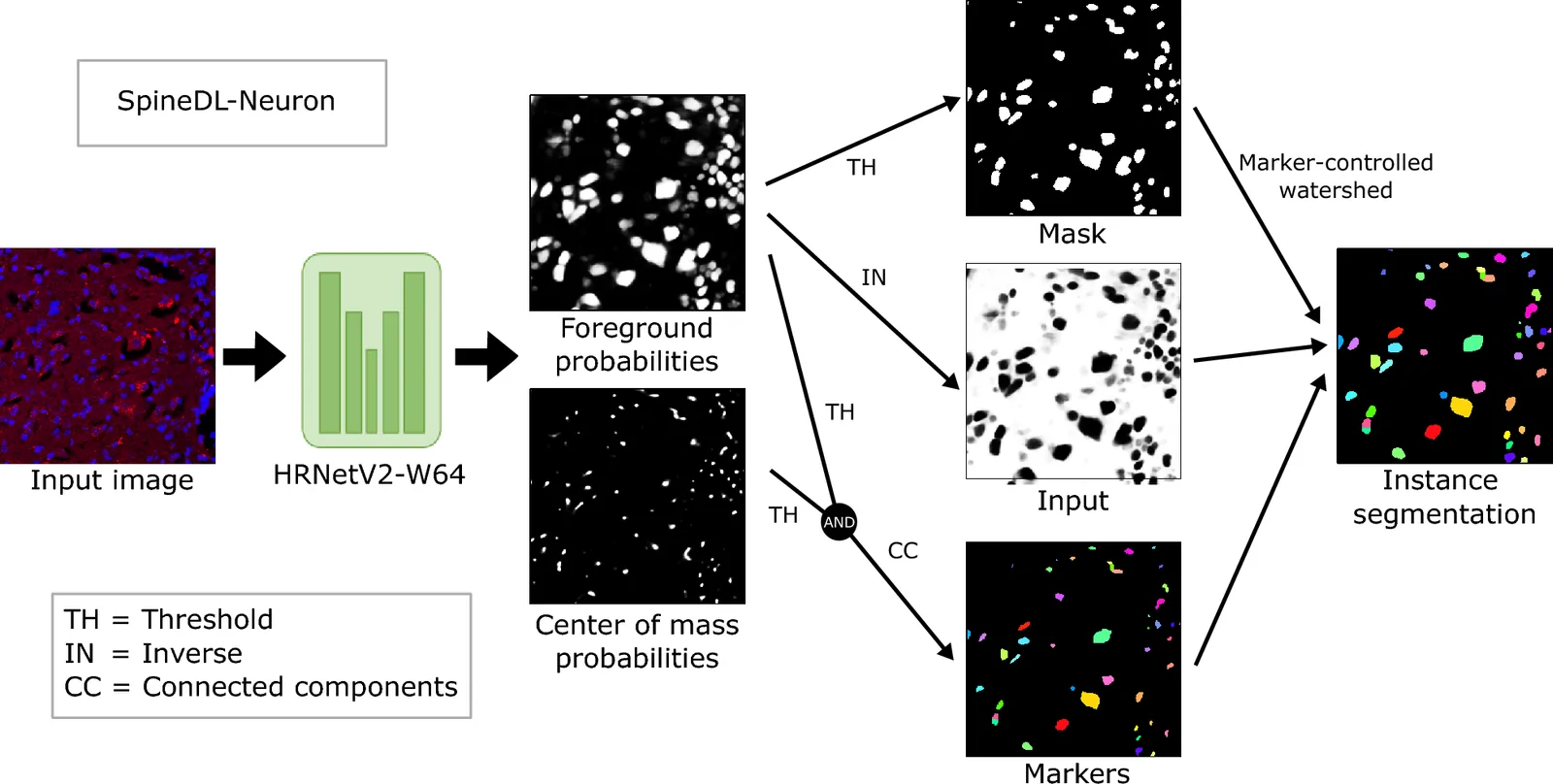
Processing pipeline of our proposed DNN for neuron identification. The DNN predicts foreground and center of mass probabilities that are fused to create three inputs for a marker-controlled watershed ^34^ to produce individual instances.

Each DNN follows a specific configuration tailored for each of the tasks as is described as follows:

**SpineDL-neuron for neuron identification**. To specifically address the task of neuron identification, i.e. an instance segmentation task, we employed a bottom-up approach inspired by the method described in^33^. The DNN architecture is trained to simultaneously predict two outputs: a binary foreground segmentation mask (F) and a neuron soma (instance hereafter) center of mass (M), which can be referred to ‘FM’. After prediction, both outputs are thresholded and combined to enhance object delineation. A connected components algorithm is then applied to the combined mask to identify discrete, non-overlapping neuron instance seeds. These seeds are subsequently used as markers in a marker-controlled watershed algorithm^34^, which incorporates three key components: (1) the inverted foreground probability map as the input image (defining the topographic surface), (2) the instance seeds as the marker image (indicating the initiation points of the flooding process), and (3) a binarized version of the foreground mask (B) as the constraint mask (limiting the extent of region expansion). This integrated pipeline enables the effective segmentation of individual neuron instances. A visual representation of the process is provided in Fig. 3.

**SpineDL-structure for anatomical structure segmentation**. Specifically designed for a semantic task, this DNN accurately segments five classes corresponding to key anatomical structures of the spinal cord, including the background, gray matter, white matter, ependyma, and damaged regions such as lesions or imaging artifacts.

### Evaluation metrics

Given that the two DNNs address distinct tasks, semantic segmentation in the case of SpineDL-Structure, and instance segmentation for SpineDL-Neuron, the evaluation protocols also differ accordingly.

#### SpineDL-Neuron for neuron identification

For each of the 10 images in the test set, the five manual annotation sessions were aggregated, and SCI researcher clicks were matched across sessions using a linear sum assignment based on spatial distance. This procedure does not alter any ground truth (GT) coordinates; it serves only to merge clicks from different SCI researchers that refer to the same anatomical object within a small spatial tolerance. Each resulting “manual object” was assigned a class equal to the number of distinct SCI researchers who clicked it (1–5).

To evaluate the detection performance, predicted instances were matched to these GT manual objects using a strict one-to-one assignment strategy. Each predicted instance was represented by its spatial centroid, and each GT object was represented by the mean coordinate of its grouped SCI researcher clicks. A pairwise Euclidean distance matrix was computed between all prediction centroids and GT centers, and linear sum assignment was applied to determine the optimal one-to-one pairings. To introduce a spatial tolerance, an assignment was only deemed a valid detection (true positive) if the distance between the paired points was *≤ 15* px. Predicted instances that could not be assigned to any GT object within this threshold were classified as model-only detections (false positives, formerly class 0), while GT objects without a valid paired prediction were considered missed (false negatives).

#### SpineDL-Structure for anatomical structure segmentation

We used the widely adopted Intersection over Union (IoU), which measures the overlap between two instances (A, B) and can be calculated as:

1 $$\begin{aligned} \textrm{IoU}(A,B) = \frac{|A \cap B|}{|A \cup B|} \end{aligned}$$

being *∩* the intersection and *∪* the union.

### Community-oriented tools and resources

To ensure that SpineDL DNNs are accessible and reusable by the scientific community, and in line with the best practices advocated by BiaPy^21^, two comprehensive tutorials have been developed for each workflow, i.e. SpineDL-Structure and SpineDL-Neuron, detailing how the DNNs can be reused in other works.

In addition, as BiaPy is a community partner of the BioImage Model Zoo^35^, the DNNs have also been published on that platform, enabling seamless integration with other tools and facilitating their rapid adoption in diverse bioimage analysis applications. Specifically, SpineDL-Structure model can be found ‘greedy-deer’ and SpineDL-Neuron as ‘proactive-snail’.

## Results

### Neuron segmentation results

We evaluated the proposed SpineDL-Neuron model (employing the HRNetV2-W64 backbone) against several state-of-the-art architectures and established baselines. These include U-Net^36,37^, Residual U-Net^37,38^, Attention U-Net^37,39^, UNETR^40^, and HRNetV2-W48^29^, as well as the widely used instance segmentation frameworks StarDist^19^ and Cellpose^41^ (v4.0.9). Additionally, we compared our approach against the proprietary Olympus CellSense Dimensions software, utilizing the specific network generated in a previous study^6^. To ensure a fair and rigorous comparison, a consistent training configuration was maintained across all models implemented within the BiaPy framework. For StarDist and Cellpose, we utilized their native default settings but aligned key training hyperparameters—specifically the data augmentation strategies, optimizer, learning rate, batch size, and number of epochs—as closely as possible with the standardized BiaPy configuration.

**Table 1:** Matching metrics between manual annotations and state-of-the-art models. Results cover precision, recall and F1 scores, with standard deviations reflecting inter-image variability. Consensus classes (1–5) denote the minimum number of expert sessions required to validate a neuron point. At a given threshold, True Positives are detections matching valid manual points; False Positives are detections matching sub-threshold consensus points or background; and False Negatives are undetected valid points. Notably, at Class 3—representing a balanced criterion that is neither too restrictive nor too loose—the proposed HRNetV2-W64 network (SpineDL-Neuron model here developed) achieves the highest performance. Models are ranked based on their F1-score at Class 3, representing the optimal consensus trade-off. Cellpose*†* denotes the fine-tuned Cellpose generalist model.

	Class 1			Class 2			Class 3		
	Precision	Recall	F1	Precision	Recall	F1	Precision	Recall	F1
Cellpose	*0.09 ± 0.07*	*0.91 ± 0.06*	*0.15 ± 0.11*	*0.07 ± 0.06*	*0.95 ± 0.05*	*0.12 ± 0.11*	*0.06 ± 0.06*	*0.97 ± 0.05*	*0.10 ± 0.10*
Stardist	*0.56 ± 0.30*	*0.38 ± 0.25*	*0.41 ± 0.23*	*0.48 ± 0.32*	*0.51 ± 0.29*	*0.41 ± 0.26*	*0.44 ± 0.33*	*0.59 ± 0.30*	*0.40 ± 0.28*
UNETR	*0.59 ± 0.21*	*0.54 ± 0.26*	*0.54 ± 0.24*	*0.48 ± 0.24*	*0.67 ± 0.22*	*0.54 ± 0.24*	*0.41 ± 0.24*	*0.75 ± 0.20*	*0.50 ± 0.26*
CellSense	*0.69 ± 0.20*	*0.43 ± 0.26*	*0.29 ± 0.18*	*0.59 ± 0.27*	*0.58 ± 0.24*	*0.28 ± 0.18*	*0.52 ± 0.29*	*0.68 ± 0.23*	*0.55 ± 0.24*
Residual U-Net	*0.74 ± 0.21*	*0.51 ± 0.25*	*0.57 ± 0.24*	*0.61 ± 0.27*	*0.66 ± 0.23*	*0.62 ± 0.25*	*0.52 ± 0.29*	*0.72 ± 0.26*	*0.58 ± 0.29*
Attention U-Net	*0.76 ± 0.18*	*0.53 ± 0.28*	*0.57 ± 0.28*	*0.59 ± 0.25*	*0.68 ± 0.27*	*0.61 ± 0.27*	*0.51 ± 0.25*	*0.76 ± 0.26*	*0.58 ± 0.27*
Cellpose *†*	*0.77 ± 0.20*	*0.48 ± 0.26*	*0.57 ± 0.25*	*0.63 ± 0.28*	*0.63 ± 0.24*	*0.61 ± 0.25*	*0.55 ± 0.30*	*0.72 ± 0.24*	*0.58 ± 0.28*
U-Net	*0.74 ± 0.21*	*0.47 ± 0.27*	*0.54 ± 0.27*	*0.62 ± 0.26*	*0.61 ± 0.25*	*0.60 ± 0.25*	*0.53 ± 0.27*	*0.70 ± 0.23*	*0.59 ± 0.26*
HRNetV2-W48	*0.75 ± 0.16*	*0.49 ± 0.28*	*0.56 ± 0.27*	*0.61 ± 0.24*	*0.62 ± 0.26*	*0.61 ± 0.24*	*0.52 ± 0.26*	*0.70 ± 0.25*	*0.59 ± 0.26*
**HRNetV2-W64**	*0.75 ± 0.19*	*0.46 ± 0.28*	*0.54 ± 0.28*	*0.63 ± 0.25*	*0.59 ± 0.26*	*0.60 ± 0.26*	*0.54 ± 0.28*	*0.66 ± 0.29*	*0.65 ± 0.21*

	Class 4			Class 5					
	Precision	Recall	F1	Precision	Recall	F1			
Cellpose	*0.05 ± 0.05*	*0.98 ± 0.06*	*0.09 ± 0.09*	*0.03 ± 0.04*	*0.97 ± 0.09*	*0.06 ± 0.06*			
Stardist	*0.39 ± 0.30*	*0.67 ± 0.30*	*0.39 ± 0.29*	*0.27 ± 0.24*	*0.70 ± 0.28*	*0.32 ± 0.26*			
UNETR	*0.33 ± 0.21*	*0.82 ± 0.16*	*0.44 ± 0.25*	*0.21 ± 0.16*	*0.85 ± 0.18*	*0.32 ± 0.21*			
CellSense	*0.43 ± 0.28*	*0.76 ± 0.21*	*0.50 ± 0.25*	*0.30 ± 0.23*	*0.83 ± 0.18*	*0.39 ± 0.24*			
Residual U-Net	*0.42 ± 0.26*	*0.79 ± 0.29*	*0.58 ± 0.22*	*0.27 ± 0.19*	*0.82 ± 0.30*	*0.43 ± 0.22*			
Attention U-Net	*0.40 ± 0.25*	*0.81 ± 0.29*	*0.56 ± 0.24*	*0.26 ± 0.19*	*0.85 ± 0.30*	*0.41 ± 0.22*			
Cellpose *†*	*0.45 ± 0.28*	*0.81 ± 0.20*	*0.52 ± 0.27*	*0.30 ± 0.21*	*0.86 ± 0.20*	*0.40 ± 0.25*			
U-Net	*0.44 ± 0.25*	*0.76 ± 0.24*	*0.54 ± 0.27*	*0.28 ± 0.18*	*0.82 ± 0.23*	*0.40 ± 0.23*			
HRNetV2-W48	*0.43 ± 0.24*	*0.78 ± 0.24*	*0.54 ± 0.25*	*0.28 ± 0.18*	*0.84 ± 0.24*	*0.40 ± 0.23*			
**HRNetV2-W64**	*0.44 ± 0.25*	*0.74 ± 0.29*	*0.60 ± 0.20*	*0.28 ± 0.19*	*0.79 ± 0.32*	*0.45 ± 0.20*			

**Fig. 4:**
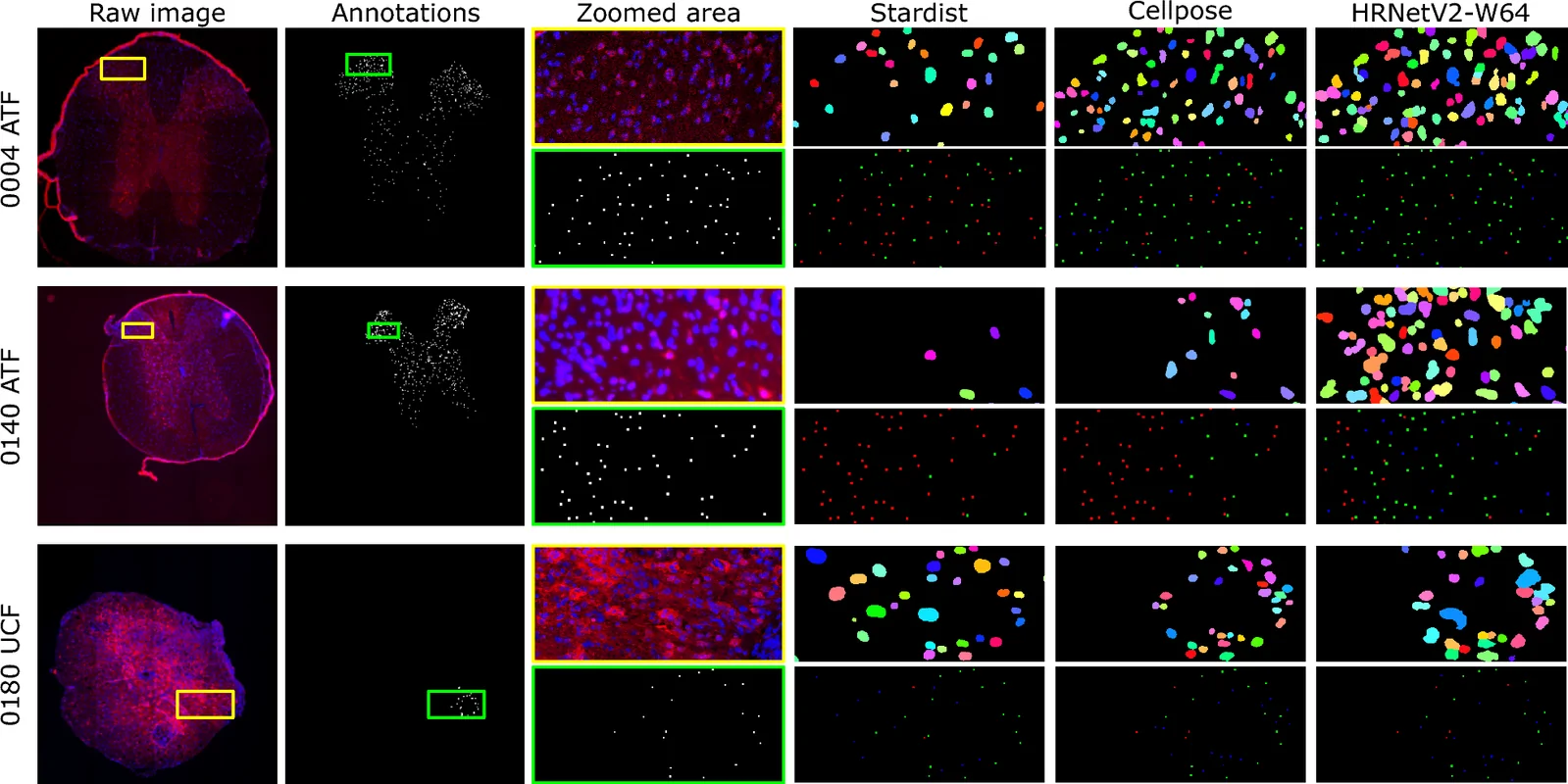
Qualitative evaluation of neuron segmentation. This figure visualizes the performance of the proposed SpineDL-Neuron (HRNetV2-W64) architecture alongside current state-of-the-art baselines. Ground truth (GT) annotations correspond to the Class 3 consensus, selected as an optimally balanced validation criterion that is neither too restrictive nor too permissive. Detection outcomes are color-coded to reflect matching metrics: green indicates true positives (correctly detected cells), blue indicates false positives (spurious model predictions lacking a valid GT match), and red indicates false negatives (valid GT cells missed by the model).

To quantitatively assess model performance, we utilized a matching strategy based on distance evaluated across five SCI researcher consensus classes (Classes 1–5). These classes define the number of independent SCI researcher sessions that annotated a ground-truth neuron point; consequently, detections matching these valid points are scored as True Positives, while unmatched predictions or those hitting sub-threshold consensus points are penalized as False Positives. Table 1 details the precision, recall, and F1-scores for all evaluated models, ranked by their F1-score at consensus Class 3. We focus on Class 3 as it represents an optimal, balanced criterion that is neither too restrictive nor too permissive. Notably, the proposed SpineDL-Neuron (HRNetV2-W64) achieves the higher overall performance over the state-of-the-art baselines at this threshold. To ensure a comprehensive and fair comparison, these baselines explicitly distinguish between the off-the-shelf Cellpose generalist model “cyto3” (denoted simply as Cellpose) and a version fine-tuned specifically on our training data (denoted as Cellpose$^\dagger$). The reported standard deviations reflect inter-image variability, underscoring the stability of the proposed architecture across diverse samples. A qualitative comparison of the state-of-the-art models with our proposed SpineDL-Neuron model (HRNetV2-W64) is depicted in Fig. 4.

To further establish a fair baseline across the evaluated methods, the confidence threshold for the center of mass (M) channel—used to identify candidate seeds in the bottom-up segmentation pipeline—was fixed at 0.5 for the proposed SpineDL-Neuron model. For the final segmentation, the foreground constraint threshold limiting region growth during the watershed step was set to 0.9 following qualitative inspection. Notably, the BiaPy framework also incorporates an automated threshold-estimation mechanism, ensuring robust, out-of-the-box performance across diverse datasets while preserving the option for manual fine-tuning.

### Structure segmentation results

**Fig. 5:**
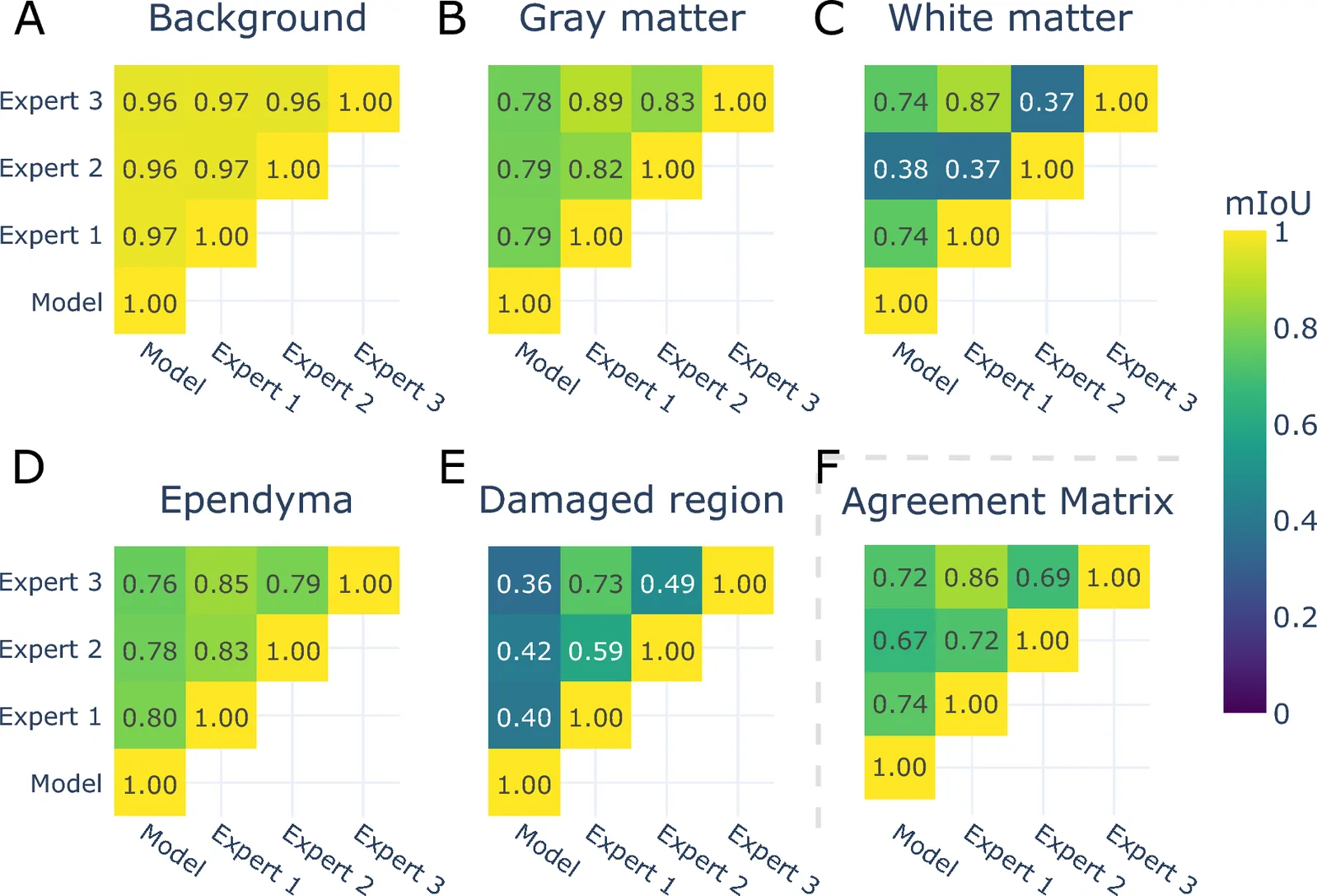
Agreement matrices (dataset-level IoU). (**A–E**) Per-class IoU agreement matrices between the SpineDL-Structure and all SCI researcher annotations. High agreement is observed for well-defined structures (gray matter, ependimum), while lower scores in the damaged region reflect higher annotation uncertainty. (**F**) Overall pairwise IoU among SpineDL-Structure and SCI researchers. The model–expert agreement (*≈ 0.71*) matches inter-expert consistency, ranging from 0.69 to 0.86, indicating than, overall, the SpineDL-Structure performs comparably to a human annotator.

The agreement matrices in Fig. 5 provide detailed overall and per-class intersection-over-union (IoU) scores between the SpineDL-Structure predictions and each SCI researcher annotation, averaged over all test images, hereafter referred to as the mean IoU (mIoU).

Overall, the SpineDL-Structure achieves an average IoU of approximately 0.71, comparable to the consistency observed among the human SCI researchers themselves. The lowest expert–expert agreement is 0.69 (Expert 3 vs Expert 2), while the highest reaches 0.86 (Expert 1 vs Expert 3), defining realistic lower and upper bounds of human consistency for this task. As the model’s performance falls within this human range, it can be interpreted as behaving similarly to an additional SCI researcher annotator. This range also highlights the intrinsic difficulty of the segmentation problem, as perfect consensus is rare even among specialists.

At the class level, the results confirm that segmentation difficulty varies markedly across anatomical structures. High agreement is observed for well-defined regions such as the spinal cord section (as opposed to background), gray matter, and ependyma, all of which present consistent boundaries and clear visual definitions across annotators and the model. In contrast, the damaged region exhibits substantially lower IoU values, even among human SCI researchers, reflecting its subjective and irregular nature. Intermediate agreement levels are observed for white matter, whose distinction from damaged tissue is often ambiguous in injured sections. This pattern indicates that both the model and SCI researchers reliably segment well-defined anatomical structures, whereas regions with irregular or ill-defined boundaries remain challenging. These discrepancies stem partly from inherent annotation uncertainty, but crucially, they also reflect a methodological limitation: the NeuN/DAPI staining protocol is not specifically designed to delineate tissue damage. Because these markers lack specific indicators for injury, capturing the exact boundaries of damaged areas is highly ambiguous for human annotators, and even more so for the model, which currently falls behind SCI researcher consensus in these zones. The damaged tissue class was nevertheless retained to ensure complete anatomical classification, though we acknowledge this limitation and emphasize it is not intended as an isolated quantitative endpoint.

We evaluated the proposed SpineDL-Structure model (utilizing the HRNetV2-W64 backbone) against several state-of-the-art architectures, including U-Net^36,37^, Residual U-Net^37,38^, Attention U-Net^37,39^, UNETR^40^, HRNetV2-W48^29^, and nnU-Net^41^ (v2.6.4). To ensure a fair comparison, a consistent configuration was maintained across all models, with the exception of nnU-Net, which utilizes an automated framework to self-configure based on dataset characteristics.

**Fig. 6:**
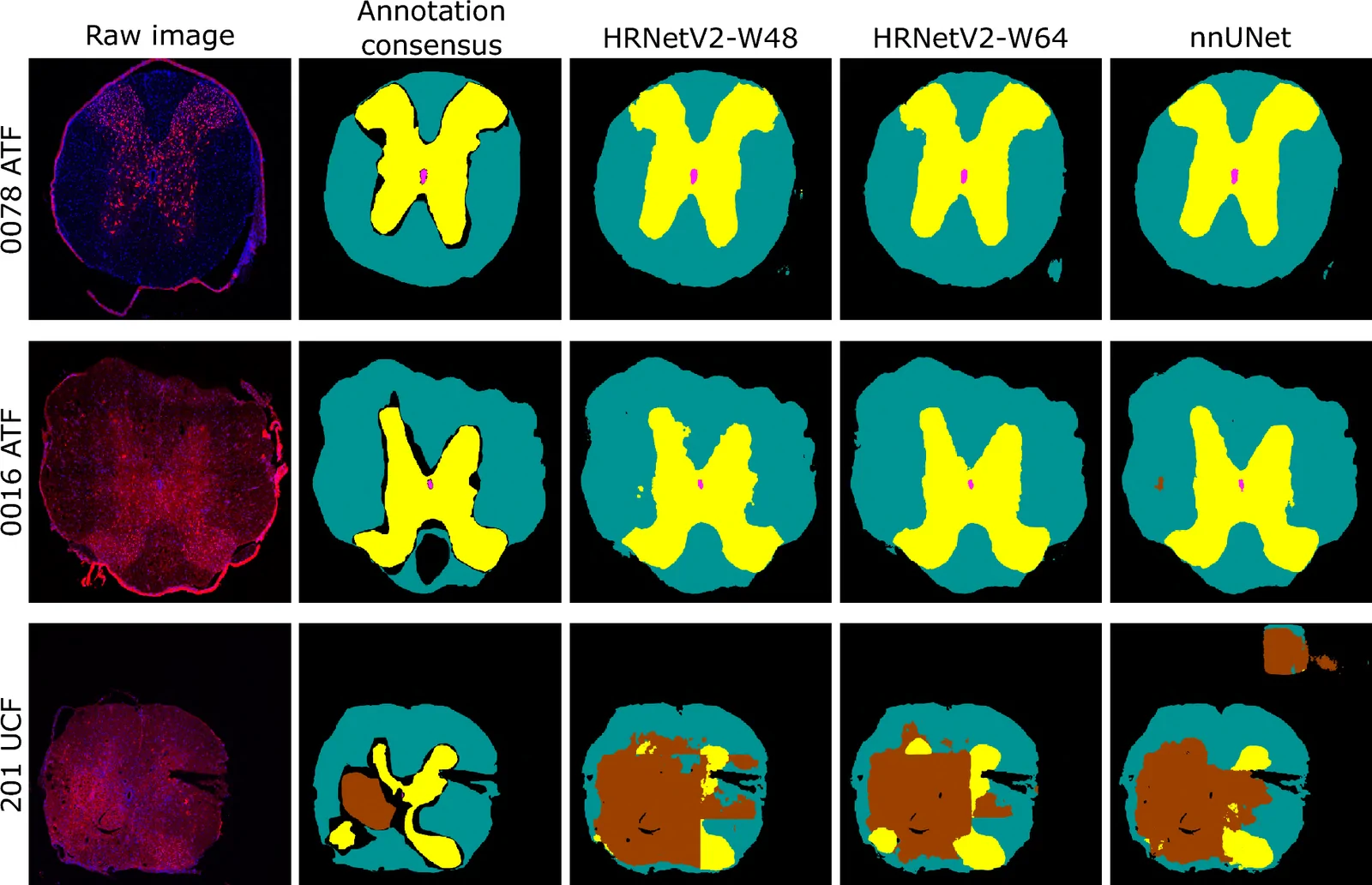
Qualitative comparison of state-of-the-art models vs unanimous-consensus GT. Examples showing unanimous-consensus ground truth (left) and the state-of-the-art model predictions in three images of differing difficulty. The visualization illustrates that most discrepancies occur along structure boundaries and within damaged regions, consistent with their higher annotation uncertainty.

Initially, we assessed class-level agreement metrics against a unanimous-consensus ground truth, constructed by merging all SCI researcher annotations and retaining only pixels with full agreement (Table 2). By isolating the most unambiguous regions of the tissue, this evaluation establishes a baseline for model performance under optimal conditions. In this favorable setting, the proposed SpineDL-Structure and nnU-Net emerged as the top-performing models, both achieving an mIoU of 0.81. However, while both models demonstrated high accuracy in segmenting well-defined structures like gray matter, white matter, and the ependymal canal, performance remained notably lower in damaged regions. These findings reveal that while the models are highly reliable on clear anatomical boundaries, they still struggle with the inherent ambiguity of pathological features, remaining below SCI researcher-level consensus in these areas. A qualitative comparison of the three top-performing models (ranked by average IoU) is presented in Fig. 6. Notably, nnU-Net occasionally misclassifies large patches of noisy background as tissue. A similar, yet more pronounced, artifactual behavior was observed in Attention U-Net, accounting for the diminished white matter performance metrics reported in Table 2.

However, when evaluating the algorithms against natural human variability, a different perspective emerges. A comparative analysis of each model’s performance relative to individual SCI researcher annotations is provided in Table 3. Here, models operating within the inter-expert agreement boundaries—defined by the lower and upper bounds of human variability—are highlighted in bold. Overall, the top-performing models successfully fall within the boundaries established by Expert 1 and Expert 3. This demonstrates that, despite minor discrepancies with the unanimous ground truth, the leading architectures achieve a robust approximation of human-level annotation consistency in general practice.

**Table 2:** State-of-the-art models vs unanimous-consensus GT. Per-class IoU between state-of-the-art model predictions and the unanimous-consensus ground truth, built from pixels where all experts agreed. The models are ranked based on the average IoU. HRNetV2-W64, marked in bold, corresponds to the SpineDL-Structure model here developed.

	Gray matter	White matter	Ependyma	Damaged region	Average
Attention U-Net	0.86	0.00	0.86	0.47	0.55
UNETR	0.84	0.73	0.84	0.48	0.72
U-Net	0.83	0.77	0.85	0.43	0.72
Residual U-Net	0.87	0.86	0.85	0.47	0.76
HRNetV2-W48	0.89	0.91	0.89	0.49	0.79
**HRNetV2-W64**	0.90	0.93	0.90	0.50	0.81
nnUNet	0.93	0.94	0.87	0.48	0.81

**Table 3:** Agreement between state-of-the-art models and expert annotations measured with IoU. The values are averaged between all classes. Inter-expert agreement establishes a performance boundary, with the lower and upper bounds highlighted in italics and bolditalics, respectively. Model performances that fall within this expert-defined range are indicated in bold. HRNetV2-W64, marked in bold, corresponds to the SpineDL-Structure model here developed.

	Expert 1	Expert 2	Expert 3
Attention U-Net	0.58	0.55	0.57
UNETR	0.69	0.60	0.68
U-Net	0.71	0.61	0.70
Residual U-Net	0.71	0.62	**0.70**
HRNetV2-W48	**0.75**	0.66	**0.73**
**HRNetV2-W64**	**0.74**	0.67	**0.72**
nnUNet	**0.76**	0.64	**0.74**
Expert 1	1.00	*0.72*	*0.86*
Expert 2	***0.72***	1.00	***0.69***
Expert 3	*0.86*	***0.69***	1.00

## Discussion

In a previous study^6^, we demonstrated that a trained neural network can outperform manual and threshold-based identification methods in segmenting neuron somas from undamaged and damaged spinal cord sections stained with the neuronal marker NeuN and the nuclear marker DAPI. The network, developed using the Olympus software CellSense Dimensions, produced predictions highly consistent with manual annotations, overcoming limitations of reproducibility, repeatability, and time: one of the main constraints in large-scale image analysis. This tool enabled the rapid and efficient assessment of neuronal distribution across dozens of sections from multiple control and SCI individuals with or without antiapoptotic drug treatment.

From these results, two main directions for further development were identified: (1) the need for an open, non-proprietary neural network for neuronal segmentation; and (2) the improvement of image registration systems toward fully automated workflows. The present study addresses both objectives. First, we developed SpineDL-Neuron, an open-source, scalable, and fully documented deep learning model for instance segmentation of neurons, designed to comply with FAIR principles and open science standards. Second, we generated SpineDL-Structure, a second DL model for semantic segmentation of the main anatomical structures of the spinal cord, aimed at facilitating image registration.

The SpineDL-Neuron module, quantitative evaluations (Table 1) reveal that our proposed architecture (HRNetV2-W64) achieves the highest performance among all tested models at the optimal consensus criterion (Class 3). Notably, it outperforms established instance segmentation frameworks such as StarDist and Cellpose. We attribute StarDist’s lower comparative performance to its inherent difficulty in modeling and representing the non-convex shapes of certain instances during training. Furthermore, our proposed model surpassed even a data-specific, fine-tuned version of Cellpose’s generalist “cyto3” model (denoted as Cellpose*†*). We believe we achieve this superior performance—even with simpler architectures like U-Net—because our proposed method utilizes a straightforward foreground probability and center-of-mass representation (referred as ’FM’ in this manuscript). This approach is easier for the network to learn and computationally lighter than the complex flow representations required by Cellpose. It is also important to underline that our models were trained entirely from scratch, whereas Cellpose*†* leveraged extensive pre-training on large datasets, giving the high performance of our method even greater merit.

Moreover, while the model exhibits some susceptibility to artifacts in lesion zones, these can be efficiently removed by masking the outputs with the gray matter predictions generated by the SpineDL-Structure model. This synergy between the two models highlights a technical superiority over previous iterations reliant on proprietary software like cellSens Dimensions^6^. Furthermore, by employing a true instance segmentation approach, SpineDL-Neuron accurately captures individual neuronal somas, bypassing the need for the complex post-processing steps typically required by standard binary semantic segmentation maps.

To the best of our knowledge, SpineDL-Structure represents the first model of its kind trained on fluorescence images of injured spinal cords. Its development posed particular challenges, as the staining protocol used (immunofluorescence against NeuN plus DAPI nuclear staining) was optimized for neuronal nuclear labeling rather than for distinguishing gray and white matter or identifying damaged areas. Despite these challenges, the results presented in Table 3 demonstrate that multiple state-of-the-art architectures, including our proposed model, achieved performance metrics that fall strictly within the inter-expert agreement boundaries (specifically between the lower and upper bounds established by Expert 1 and Expert 3). Reaching this “human-level” accuracy suggests that these models are reliable enough to be considered as supplementary, automated annotators. We hypothesize that once this human-level baseline is achieved, any remaining variability in the metrics are primarily driven by inherent boundary inconsistencies and annotation uncertainty rather than fundamental model limitations. Accurate identification of these regions is especially relevant for image registration, as they provide homologous reference points between target and reference sections. Future work should address these limitations by incorporating additional immunofluorescent markers associated with tissue structure and damage (e.g., MBP, Iba1, GFAP), enabling more consistent and biologically grounded annotations. Training on such multimodal data could improve robustness in pathological regions and be extended to cell-type-specific markers for identifying neuronal subpopulations, ultimately advancing SpineDL toward a more fine-grained characterization of spinal cord heterogeneity.

Although HRNetV2-W64 is selected as the recommended SpineDL-Structure model, we acknowledge that nnU-Net achieved essentially equivalent performance in our benchmark, including the same mIoU against the unanimous-consensus reference annotations and very similar agreement with individual experts. Thus, nnU-Net represents an equally valid alternative for users who prefer a self-configuring segmentation framework. Our decision to release HRNetV2-W64 as the default SpineDL-Structure model is primarily motivated by practical and ecosystem-level considerations rather than by a clear superiority in accuracy. In particular, HRNetV2-W64 is fully integrated within the BiaPy-based SpineDL workflow, facilitating reproducible training, inference, fine-tuning, and deployment using the same software environment as SpineDL-Neuron. This unified implementation lowers the barrier for users in the SCI histology community, simplifies maintenance of the released pipeline, and provides a more flexible basis for future extensions, including joint or multi-task segmentation strategies combining anatomical structures and cellular instances. Therefore, while HRNetV2-W64 is proposed as the reference model within the SpineDL framework, nnU-Net remains a strong and appropriate option for users whose workflows are already centered on self-configuring pipelines.

Importantly, the iterative annotation strategy adopted in this work–where expert corrections refine DNN predictions–follows established “data engine” methodologies for dataset construction in the absence of ground truth (e.g., SAM ^27^, CartoCell ^28^). However, this paradigm inherently carries a risk of confirmation bias, as annotators may become anchored to model predictions, particularly in ambiguous regions such as damaged tissue where boundaries are not well defined. This effect can lead to a progressive alignment between annotations and model outputs rather than an independent approximation of ground truth ^42,43^. While the targeted inclusion of out-of-distribution samples helps mitigate this limitation by exposing the model to broader variability, the potential bias should be considered when interpreting performance gains across iterations. Crucially, the strict isolation of the test set throughout the process ensures that the reported evaluation metrics remain unbiased and reflect true generalization.

Overall, this work represents a significant advance toward fully automated workflows for histological image analysis, enabling large-scale, systematic studies of neuronal distribution in spinal cord injury (SCI). Unlike closed commercial platforms, the SpineDL framework is strictly aligned with FAIR principles and open science standards^44^. Implemented within the BiaPy framework, the pipeline is fully reproducible and easily retrainable to adapt to specific experimental conditions. The public release of the code, trained models, and the expertly annotated SpineDL dataset provides a transparent, highly accessible foundation for the broader scientific community to validate and extend computational histopathology in SCI.

Building on this foundation, several lines of future development can further enhance the applicability and impact of SpineDL. Although the present work focuses on the mouse spinal cord based on a training dataset that incorporates substantial variability of histological conditions, testing the models in rat sections, a widely used and translationally relevant SCI model, will allow us to evaluate the robustness of the segmentation pipeline under differences in tissue size, cytoarchitecture, staining patterns, and injury morphologies. Such validation will help determine the generalizability of SpineDL across species and experimental conditions, and may ultimately support its extension to other animal models or human post-mortem samples.

A second major direction involves the development of a 3D reconstruction pipeline for the spinal cord. The anatomical segmentations produced by SpineDL-Structure will serve as anchors for accurate image registration and integration into 3D anatomical atlases, such as the one developed by^45^ for volumetric mapping of neuronal survival. Coupling SpineDL-derived segmentations with these reconstruction methods would enable the generation of spatially normalized representations of neuron distribution following SCI, thereby facilitating analyses of its relationship with injury progression or functional deficits.

## Data Availability

The SpineDL dataset is available in the Zenodo repository (https://zenodo.org/records/17829532) under the CC BY 4.0 license. Additional metadata, raw images, and information on the subjects included in this study are available in the NeuroCluedo OSF repository (https://osf.io/n32z9/overview), also under the CC BY 4.0 license.

## Code availability

The experiments were performed using the open-source BiaPy library ^21^, available on GitHub. To facilitate reuse and reproducibility, we developed two comprehensive tutorials: one for the SpineDL-Structure workflow and another for the SpineDL-Neuron workflow. In addition, both trained models have been published in the BioImage Model Zoo. The SpineDL-Structure model is available as “greedy-deer”, and the SpineDL-Neuron model as “proactive-snail”.
